# Transformative learning through participation: experiences at a rural clinical training site in South Africa

**DOI:** 10.1186/s12909-022-03233-w

**Published:** 2022-03-16

**Authors:** Jana Müller, Cameron Reardon, Francois Coetzee, Juanita Bester, Kopano Dube, Susan Hanekom, Elmarize du Plessis, Ian Couper

**Affiliations:** 1 Faculty of Medicine and Health Sciences, Ukwanda Centre for Rural Health, Stellenbosch University, Cape Town, South Africa; 2 Physiotherapy, Faculty of Medicine and Health Sciences, Stellenbosch University, Cape Town, South Africa; 3 Occupational Therapy, Faculty of Medicine and Health Sciences, Stellenbosch University, Cape Town, South Africa; 4 Speech Language and Hearing Therapy, Dr Harry Surtie Hospital, Northern Cape Department of Health, Upington, Northern Cape Province South Africa

**Keywords:** Rural health, Undergraduate, Health professions education, Distributed training, Transformative learning

## Abstract

**Background:**

Distributed training has been cited as an opportunity that offers transformative learning experiences in preparing a future workforce to address local needs. For this reason, rural and longitudinal placements are increasingly being adopted by medical schools across the world. Place, participation and person are considered integral in the process of transformation of medical students into responsive graduates on the distributed platform. This article aims to explore the experiences and perceptions of student learning on a rural training platform in South Africa while considering the interrelation between person, place and participation as a process of transformation to becoming a health care professional. The research forms part of a 5-year longitudinal case study, initiated in 2019 to explore a university-rural hospital collaboration on students, staff and the local health care system.

**Methods:**

Data was collected using interviews and surveys from 63 purposively selected and consenting participants between January and November of 2019. All qualitative data were inductively analysed using an interpretivist approach to thematic analysis for the purposes of this article. All quantitative data was analysed descriptively using Microsoft Excel. Ethics and permission for this research was granted by the Stellenbosch University Human Research Ethics Committee, the Undergraduate Students Programme Committee and the Northern Cape Department of Health, South Africa.

**Findings:**

Four themes, namely: authenticity of context; participation in a community of practice and social activities; supervision and reflection; and distance support were extracted from the data. These findings contribute to the theory of transformative learning on the distributed platform by expanding on the interrelationship of person, place and participation, specifically as it relates to participation within various communities and practices. The value of active participation in reflection and supervision, distance academic support and social support systems are explored.

**Conclusions:**

The three dimensions and interrelationship of person, place and participation in the process of transformative learning on the rural training platform can be further unpacked by exploring the types of participation that have facilitated student learning in this research context. Participation in interprofessional teams; supervision, reflection and distance support appear to be the most crucial elements during this transformative learning process.

## Introduction

The global shortage of health care professionals in rural primary health care (PHC) environments has been an important motivator in the development of several distributed training interventions in undergraduate health professions education. Based on the understanding that the context of training influences future practice location [1, 2], the distributed training interventions being applied to address this disparity include rural rotations, community-based training and longitudinal clerkships [3–6]. Distributed training has been cited as an opportunity that offers learning experiences that are potentially transformative, producing the kind of twenty-first century graduates that health professions schools have been challenged to produce [7] and enabling a future workforce to address local needs [8, 9]. Distributed rural models of training have been associated with positive learning experiences and have been found to increase intent to practice in rural locations [4, 6]. Rural rotations and longitudinal rural placements are therefore increasingly being adopted by medical schools across the world [5, 10, 11].

Student agency and the ability to guide their own learning have been identified as crucial to the success of clinical learning away from the academic home [12]. Van Schalkwyk et al. (2014) posits that the interaction between place, participation and person are integral to the transformation of medical students training on rural platforms into professional doctors in practice [13]. Understanding the way in which students learn through exposure to new contexts on the distributed training platform can be better promoted when considering transformative learning theory. This pedagogy supports an evolving process of critical reflection and introspection, where an individual undergoes a fundamental change in their thinking and actions based on exposure to a disorienting dilemma [14]. Critical reflection of students’ experiences on the distributed training platform is one of the ways in which students and graduates have been shown to develop the capacity for continuous adaptation and awareness, making them better prepared to work in the ever-changing landscape of health care during their professional lifetime [14, 15].

Student learning experiences away from the academic center can be complicated by distance, logistics, information technology and communication access as well as the limited availability, capacity and expertise of academic coordinators and clinical facilitators on the platform [12]. Supervision and training of students in the health professions may also be a challenging new responsibility for already busy clinicians based at distributed training sites. Student agency and the ability to guide their own learning have been identified as crucial to the success of student clinical learning away from the academic home [12].

In South Africa, the need for competent professional graduates who can practice with minimal supervision or support is emphasised by the disparities in terms of the distribution of qualified and senior health care professionals in rural areas [16]. This is especially true with public health care services in rural South Africa, which are significantly under-resourced in terms of the number of health care workers employed to address community needs. These same facilities need to accommodate new graduates who are required to complete a compulsory year of community service [17].

There are also the complexities of language and cultural differences across the country that can affect the interaction between patient and health professional. These communication challenges require graduates to adapt and respond appropriately. Therefore, early exposure to a variety of communities and cultures is necessary to ensure that students and graduates develop the appropriate skills to adapt and respond to challenges related to language and cultural barriers. Placing students in remote sites and exposing them to new environments far from their support structures and academic base for extended periods of time, adds an additional dimension to student learning. Often this abrupt and comprehensive change of learning environment and life experiences may require students to allow for a period of adapting to the context of learning.

Stellenbosch University’s Faculty of Medicine and Health Sciences (FMHS) has increasingly expanded its presence on the distributed clinical platform to train students in the health care professions. Positive learning experiences reported by students and health services outside the tertiary hospital learning environment has catalysed the expansion of distributed training sites, which has also assisted in accommodating the increasing numbers of students in the undergraduate health professions programmes [18]. Although the academic home of the faculty is in Cape Town at Tygerberg Hospital, where the majority of students’ clinical training takes place, there are now multiple distributed clinical learning sites, mostly in the Western Cape Province.

In 2018, the FMHS initiated the development of a new rural distributed training site 800 km away from Tygerberg Hospital at the Dr Harry Surtie (DHS) Hospital in Upington in the Northern Cape Province [19]. DHS Hospital is a 327-bed regional hospital, which in 2018 had 240 operational beds due to staff shortages. The hospital provides specialist medical services to the entire western half of the Northern Cape, which is the largest of South Africa’s provinces. The hospital has a limited number of medical specialists, with some departments being run by experienced career medical officers or foreign-qualified doctors who do not have South African specialist registration. In 2019, short rotation rural clinical placements for final year medical, occupational therapy and physiotherapy students started on the platform. These rotations varied from 4 to 6 weeks. Longitudinal clinical placement for final year medical students for a period of 10 months ran concurrently to the short rotations. All students had prior clinical training at Tygerberg Hospital in Cape Town.

Clinical supervision of students training at DHS Hospital was dependent on clinical staff working in the private and public sector in Upington. Clinicians and students were supported by visiting academics and clinical specialists from Cape Town on a regular basis. Academic staff of the FMHS were responsible for overall programme coordination and student assessment, traveling to Upington as deemed necessary.

To determine the influence of the university-hospital collaboration on the students, staff and health system, a longitudinal research project was developed to evaluate the effect of the university’s involvement in Upington over a period of five years. This article explores student learning at DHS Hospital during 2019, based on the perceptions of stakeholders involved with the Upington distributed training platform. The aim of this article is to examine and describe the experiences and perceptions of student learning on the rural training platform, while considering the interrelation between person, place and participation as a process of transformation into becoming a professional.

## Methods

The over-arching longitudinal research study uses a descriptive embedded case study research design. Qualitative and quantitative data was collected using interviews with and surveys of 63 purposively selected and consenting participants between January and November of 2019. The details of the methodology, including interview schedule and survey details, have been described elsewhere [19]. A summary of data collection methods can be found in Table 1. The rationale for including academic coordinators, students, clinicians and supervisors in the study (see Table 1) was to explore the perceived value of the development of the Upington training platform from multiple perspectives.

**Table 1:** Data collection methods and participants for the over-arching research study

Data collection method	Participants	Number of Participants
Semi-structured individual interviews	Supervising medical clinicians	3
	Supervising rehabilitation clinicians	3
	Dr Harry Surtie Hospital facility managers	3
	Academic programme managers at Tygerberg	8
	Final year medical students undergoing longitudinal placement	4
Pre- and post-rotation surveys with open and closed ended questions distributed using Research Electronic Data Capture (REDCap™) software [20]	Short rotation final year physiotherapy students (pre/post rotation)	4
	Short rotation final year occupational therapy students (pre/post rotation)	8
	Short rotation final year medical students	10
Semi-structured focus group interviews	District health managers	5
	Nursing preceptors	3
Brief semi-structured conversations [21]	Non-supervising clinicians and administrative staff from Rehabilitation, Internal Medicine, Paediatric, Orthopaedic and Surgical departments as well as staff working in the Intensive Care Unit	12
Total participants		63

All data were anonymised prior to analysis. All qualitative data were inductively analysed using an interpretivist approach and codes were identified. Code-book thematic analysis [22] was done by all the researchers using an agreed upon code book. All quantitative data was analysed descriptively by SH, using Microsoft Excel.

Issues of trustworthiness were minimised by the triangulation of data collection (quantitative and qualitative, multiple student disciplines and rotations), and analysis: all authors participated in the qualitative data analysis process and member checking of participant interview transcripts. The authors include clinicians and academics from various health care professional programmes, from both Tygerberg and Upington, who have been involved with the development of the Upington distributed training platform since 2018.

Only individuals who provided written informed consent were included as research participants. Ethical and institutional approval was obtained from Stellenbosch University’s Faculty of Medicine and Health Sciences Human Research Ethics Committee (#N19/02/026) and the Undergraduate Programme Committee. Permission to conduct the research was also provided by the Northern Cape Department of Health.

### Findings

Four themes which pertain predominantly to students' perceived learning experiences were constructed from the qualitative data and are presented below using direct quotes from the transcripts analysed. Table 2 provides a key to the abbreviations used to represent the various stakeholder responses. Survey results (quantitative data) that were linked to these four themes are included in the theme description (embedded mixed methods design).

1. The authenticity of the learning context
2. Participation in a community of practice and social activities
3. Supervision and reflection
4. Distance support.

**Table 2:** Key to abbreviations used for stakeholder responses in the findings

Participants	Abbreviation
Dr Harry Surtie Hospital facility managers	HFM
Academic programme managers at Tygerberg	APM
Medical students undergoing longitudinal placement	MS
Short rotation physiotherapy students (pre/post rotation)	PT pre/post
Short rotation occupational therapy students (pre/post rotation)	OT pre/post
Short rotation medical students ( pre/post rotation)	MBChB pre/post
Non-supervising clinicians and staff	NSC

#### Theme 1: the authenticity of the learning context

The nature of the exposure at the site and the influence this had on students’ learning was an important contextual factor that all stakeholders considered in their reflections prior to placement. Students expressed eagerness to experience new training environments that could afford them the opportunity of “serving a different community as well as experiencing different community needs, therewith broadening my perspective on holistic healthcare.” (MBChB1pre). “I am looking forward to having a general block as well as being in a different environment as this will prepare me well for community service and my future as a physiotherapist.” (PT2pre).

One hundred percent of the short rotation students who completed the survey after their placement at Upington agreed that the setting provided insight into the cultural and social challenges of local communities. Similarly, clinicians felt that students, “Experienced maybe the true South African circumstances, as far as healthcare goes,” (HFM1) due to the nature and reality of an under-resourced hospital setting.

Academic coordinators from the medical programme, based at the University’s Tygerberg Campus, expressed concern about the clinical exposure and teaching in a less specialized hospital environment. “I do think the medical students are losing out as they are not exposed to a place like [XX] where they could get exposed to a higher level of medical training.” (APM6). “So, part of it was a concern that they were not exposed to a broad spectrum of surgical conditions that they may need to pass their exams and to be good doctors in future.” (APM3).

However, even though some students reflected on the challenges of working in a hospital that was not academic, the authentic experience of working in a resource-constrained environment allowed for a process of critical reflection. “And sometimes things happen, you're like… this is the wrong treatment, or we should do more, but you learn to think of it more critically about what you do… You don’t just see these consultants that are, wow. You see it gets rough, you see if you don't keep on learning, you're gonna be a dangerous doctor and you've seen that you need to like, challenge yourself.” (MS2).

“It can really become a good academic site, because there's a big hospital there's a lot of things if you think all the resources you need are here … I think it can be an amazing place to send students to cause they gonna… You're going to work as an intern [first year graduate] in the sense of you're gonna be like independent, you're going to think for yourself and learn to reason and all those things, but you're also gonna have someone to check on you and make sure you're on the right track.” (MS2).

Having fewer specialised departmental teams seemed to afford students the opportunity of seeing patients from all sectors with varied and multiple pathologies. “I enjoyed having exposure to different types of patients and not seeing patients in a block format, e.g. I saw a CVA [cerebrovascular accident] patient and a patient with an olecranon fracture on the same day, where otherwise you would either see patients with only neurological conditions or only orthopaedic conditions in other set-ups.” (PT2post).

Students, particularly those from the rehabilitation sciences, reflected on the wide range of clinical exposure they experienced at the site, which they may not have had elsewhere. This exposure was perceived to prepare them to be future healthcare practitioners. “A very wide range of conditions was seen, both in hospital and outpatients. I was allocated to the surgical ward, but also saw patients in the medical and paediatric wards. I also had the chance to assist my clinician with a few paediatric patients in the ICU [intensive care unit].” (PT4post).

“Hands is a domain within OT [occupational therapy] – we were exposed to hands at Dr Harry Surtie where we rarely are in the Western Cape placements. This was great. Also, the paeds [paediatrics] – we rarely see FAS [Foetal Alcohol Syndrome] in our clinical placements in the Western Cape and this was great for us to see the role it plays within a child's development.” (OT6post).

Despite these positive experiences, students critically reflected on the need for more exposure to primary health care. “The only interaction we had was with one district clinic that we have asked and arrange with our clinicians to attend. I feel the hospital was a bit isolated from the community in a sense. I feel our programme can be broadened to include more district clinics.” (PT2post) “We were not allowed to do home visits, which limited our learning experience.” (OT1post). Students not only saw the need for more primary health care experience as something that could benefit their future practice, but also as a socially accountable way of improving access to care in a human resource constrained environment. “There is definitely a need for community projects within the district … The district physio is a comm serve [doing compulsory community service] with a very high workload leading to her only being able to attend clinics once every three weeks. We as students can definitely help in this regard by being more hands and helping to see more patients in a shorter period of time.” (PT2post). “I think it would in the end, become a thing where the students can help lift like the patient burden, so that patients get better care and get better treated more quick and more efficiently.” (MS2).

#### Theme 2: participation in a community of practice and social activities

Students took a more active role in seeking out clinical learning opportunities that were available at the site. “We were given a schedule and general academic aims for the block, but the majority of the academic learning was by opportunity and in a sense, we were given the responsibility and freedom to seek our own learning opportunities. For example, I do not have a paediatric block this year, so I was able to ask my clinicians to give me more paediatric patients to see in order for me to have that exposure.” (PT4post). Thus, the learning opportunities could be individualized to suit the student’s perceived needs if they were willing to direct their learning.

Students described the benefit of interprofessional engagement that occurred at the site and how this positively influenced their learning. “We had three tuts [tutorials] with the medical students on ortho evaluation techniques for the hip, knee and shoulder, and various discussions on referrals, specific patients, X-rays, etc. in the hospital wards. This was empowering both for us [and] the medical students to understand how we can work better together in a multidisciplinary team in the future.” (PT4post).

Eighty-two per cent of the short rotation students who participated in the survey agreed or strongly agreed that ‘the clinical exposure in Upington was relevant to what I needed to know’ and 100% responded that ‘there were opportunities to interact with and learn from other health care students and professionals’. These experiences stood in contrast to the long-term medical students on the platform. “We'd have no interaction with any of the other students so ja that's also kind of feeling excluded most of the time.” (MS1).

Being given responsibility was seen to be a key factor in students’ development. “I was given co-responsibility with my clinician to help manage our allocated ward and a lot of flexibility in my time management. I was in a position where I could learn how to take more initiative to find solutions for the challenges and shortfalls in the system, e.g. referrals from the doctors, work within a multidisciplinary team approach, and learn about the referral pathways and better discharge planning.” (PT4post). “I felt I gained clinical skills as well as the confidence to manage a ward, while also managing an outpatient schedule more effectively as a physio [physiotherapist].” (PT2post).

Students responded well to feeling like they were a part of the team. “I personally found great value in being treated more like a colleague than a student by the healthcare team in the hospital for the majority of the time, especially at this point in my studies.” (PT4post).

The students perceived that their participation in the community of practice not only facilitated their own learning but also benefitted the health system. “I think the doctors have sort of like gotten used to us being around. They know what we do and all that and now … because now we want like the updated information, updated guidelines. So, then it benefits us, it benefits the doctors, it also benefits the patients because they're getting the newest, best evidence-based medicine.” (MS3).

Students placed in Upington for extended periods of time expressed concerns about social isolation. “So socially I don't know what I could suggest but, in the beginning, I did feel quite cut off like we're working with students but not really part of something.” (MS1).

The experiences of the longitudinal medical students stood in contrast to that of the shorter rotation health science students, 100% of whom noted that the hospital staff included them in their social activities. “Since there are quite a number of the members of the healthcare team that are doing their community service year, as well as final-year medical students [short rotation], we were often invited to join them in social activities after work and were welcomed into the community very quickly…. My clinical partner, [XX], joined the rugby club from the first week, and they invited us to their socials after practice…I was honestly astounded by how we were really welcomed and made part of the community in Upington since day one.” (PT4post). An interesting comment from a short rotation medical student noted the value of participation with students from other professions. “If you are outgoing and can find something to do with the allieds its nice. Otherwise, you'll be quite lonely.” (MBChB2post).

The need for proper social inclusion and support was perceived to be very important to the wellbeing of the students who were placed there for long periods, and recommendations were made by students based in Upington relating to improved social inclusion and engagement from a distance for future students. Inclusivity with their peers, whether virtual or face to face, was perceived to be an important part of coping and wellness.

“My advice [to students] would always be to remain social with the people that they're working with; that they stay in close contact with their peers and their colleagues, because these are the people that are going to feed a lot into them as they grow up outside the hospital environment. It keeps them in touch with their work, but it also keeps them in touch with their general and broader community.” (MS4).

#### Theme 3: supervision and reflection

While students enjoyed greater levels of responsibility and authentic participation as a member of the health care team, adequate academic support was important for their development. More specifically, one student noted the value of supported reflection in her own learning journey, particularly when facing challenging issues. “It definitely reassured me that we have good support and were able to report and discuss challenges/issues we faced during the block. I felt encouraged to take more initiative in finding and engaging in learning opportunities. We also discussed our experiences each week, which I personally found to be a good form of clinical reflection.” (PT4post).

The availability and willingness of clinicians to engage in student training was also a common theme that emerged, participants also mentioned feeling more confident to apply new learning and show initiative. Ninety-one per cent of the short rotation students agreed with the statement that the site facilitator/s or student supervisors were approachable and enthusiastic. “This is an amazing environment to practice your skills and the staff are more than willing to assist. Don't play it safe [with regards to learning opportunities] you can do that in Tygerberg.” (PT3post).

There was consensus among the students regarding the supportive influence local clinicians had as positive role models on their learning. This was especially true for the short rotation students. “The constructive feedback they gave us made our learning easier. Their passion for their specific fields made me sure of what field I would like to specialize in. My positive experience was definitely because of the support I received from the allied health department.” (OT6post).

“In [XX] I saw an example of the type of physio I would like to be or at least aspire to be one day. Many small acts advocating for patients, reading x-rays, following up, small details remembered from patients and many such things really stood out.” (PT2post). It was “encouraging to see how she works with and really loves her patients [Speech Therapist].” (MBChB1post).

“I had the opportunity to spend a lot of one-on-one time with my clinician, and she was readily available for questions. This boosted my confidence immensely! By observing and working with her, I gained a deeper insight on how to advocate for my patients and the need for having sound knowledge of the referral pathways and environment outside of hospital.” (PT4post).

However, too much responsibility without adequate support had the potential to create negative experiences for students, which may have impacted their learning. The expressed need for more clinical supervision and support was predominant to one group of participants. “You would literally see patients [in the clinic] and have no idea what you’re doing but this patient’s health is now in your hands … I don’t think that’s completely fair to the patient but also to us cause its hours we spend thinking we’re doing things, but we don’t know if we’re right and it’s, it's scary cause you wanna, you know, do what’s best for the patient, you don’t always know if that is what you doing, so…” (MS2).

“But I think we need to make sure that the people that are supposed to support us does support us. … it's not my job to keep [them] accountable to teach me. … I know you're responsible for your own learning, but … the people that should be in charge are in charge, and when they have meetings, it's meetings that actually mean something.” (MS2).

Concerns about the support available to students were also noted by clinicians not involved with student supervision. “I am sometimes a bit worried that they don’t have the best or no supervision or more guidance and I think that leaves them a lot to learn on their own. So, you can see they really work hard on their own, but I sometimes feel they don’t get that input that they actually should … as an SI [student intern]…” (NSC7).

“And then many times you find that the clinicians are just so overworked that they can't find time to spend with the students,” (MS3) and it was perceived that there was “less support than [for] students [placed] close to home.” (MBChB4post).

#### Theme 4: distance support

The challenges of distributed clinical placements in terms of student learning and wellness were obvious, and the support students would need was recognised early on by academic coordinators. “Even though we might send one or two students, it’s still students that we need to take responsibility for, and responsibility for probably a large extent teaching and learning but also mental health, also safety. Do they have enough food? And this is where my doubt comes in.” (APM1).

The students’ experience of participation with their academic departments was varied across departments. In addition to direct on-site support, distance support strategies were helpful to some students “Our lecturer was always available via email or WhatsApp if we required more information etc.” (PT2post). “We had a WhatsApp group, which was very effective, as well as weekly Skype calls.” (PT4post).

Some students reported a need for greater distance support, which in many cases was discipline dependant: “Like every second month they can just like check in, how are you doing, is everything alright? What do you need? But more from a place of genuine care, I guess. And then engaging us on what the other students are doing across the board. That would be good.” (MS4).

“Sometimes we feel we don’t have a lot of support from the university.” (MS1). “We missed out quite a lot of tuts [tutorials]. … And I think that's unfair because the LIM [longitudinal integrated model] people are told, you'll have tuts with consultants, but then it doesn’t happen, and then you self-study all of the work, which is scary.” (MS2).

“So, there were those times where I felt overwhelmed by the work and the responsibility and everything’s to come. And that's why I said, once again, that social support, that student support is very important, very needed. And even if it's something small. If the University or if the hospital can offer a helping hand when there are students in need, that can really make much of a change in the students.” (MS4).

Access to distance support structures using Skype, WhatsApp or Microsoft Teams was crucial to the wellbeing of students on the distributed platform. In some cases, limitations of data availability or information technology negatively affected students’ experiences and access to these necessary support structures. “And they still give us 30gigs [gigabytes] between two people to use for the month when we have five Skype tutorials each week, one Skype session which is an hour, takes about one gig” (MS3). Thirty-six per cent of short rotation students disagreed with the statement that ‘There was adequate access to information technology’. This was in contrast with the internet accessibility for students at the faculty campus in Tygerberg, where access to data was not limited. “You know which part of the thing that made it so difficult for us is … the fact that we didn't even have enough data to say, Skype our parents or maybe video, where on campus [Tygerberg] like maybe you would have used some of your internet quota for entertainment, here you can't do that so ja, just getting by that.” (MS1).

## Discussion

Students reported a significant breadth in their clinical exposure at this training site, encountering a wide variety of clinical conditions, some of which they had not previously encountered. Attributable to the generalist nature of rural practice, this breadth in exposure seems to be a common feature of rural clinical placements [4]. Encountering a greater variety of clinical conditions within a more generalist environment undoubtedly benefits the learning of students, and it is through this exposure that students develop confidence and competence in clinical management [13]. The capacity of students to reflect on their challenging learning experiences and use this to determine who they want to be as professionals contributes to the notion of a transformative learning experience. Situating clinical education within unfamiliar contexts may more readily facilitate transformative learning, as students are more likely to encounter what Mezirow termed ‘the disorienting dilemma’ in novel environments to which they are unaccustomed [23].

It is clear that transformative learning opportunities exist within this clinical training context. There are indications that, although noted as uncomfortable, students reported a transition from being observers to being contributors within clinical environments. Whether this exposure has resulted in an ‘integrated and irreversible’ way of being for these students still needs to be explored (Van Schalkwyk et al., 2019 pg. 552). However, the emerging advocacy within students’ responses to contribute to an under-resourced health care system demonstrates a new way of thinking and being for students that extends beyond clinical competence towards greater social accountability. In this regard, the wish reflected by students to be more community oriented or to expand their clinical learning activities to reach more underserved facilities within the district is most encouraging.

Reid (2011) proposes a number of ways that rural contexts potentially lend themselves to this type of transformation. One of these is that the health systems within rural contexts are more generalized, allowing for easier interaction between disciplines, thus making it easier for students to identify and interact with the system in its entirety [24]. Some students within this cohort had exposure across the health services platform, including home visits, primary care clinics and the district/regional hospital setting. However, there was an expressed desire from students to become more involved in primary health care. This system-based exposure appears to have translated into a degree of systems thinking for some students and is more likely to result in the requisite transformation that is necessary for contemporary health professionals [7].

Students described a greater sense of autonomy both in clinical practice and in directing their own learning, a phenomenon commonly reported at other rural clinical sites [13, 25, 26] and believed to result from a number of factors including greater co-operation between clinicians and students [24]. The effect being a more ‘hands-on’ exposure for students and as a result a sense of greater competence in clinical practice [27]. But while the benefits of meaningful engagement in clinical practice and greater autonomy were clearly articulated by most students, not all students experienced this positively. At least one student observed that greater autonomy without sufficient support is in fact detrimental. Safe and inclusive learning environments more readily foster transformation [28] and it is plausible that clinical environments with insufficient support may be perceived by the individual in a threatening context and thus hamper active participation and resultant transformation.

### Participation for transformation

While it is the rural context that provides the opportunities for transformation, it is the active participation of students within the community of practice at the site that is likely to translate into a new way of being for these students [13, 29]. Enabling participation at distributed sites can be complex. The authors explore opportunities that foster student participation as a means of transformative learning on the rural distributed training platform.

Important themes that were identified from this data support the notion that rural clinical learning spaces are potentially transformative learning environments. This adds to the extensive body of literature that supports the value of rural, distributed training for undergraduate health science students [9, 30]. The findings presented here reiterate important dimensions of transformative rural spaces and contribute to the notion of participation in rural contexts and the importance of participation in a community of practice [13, 29].

**Fig. 1:**
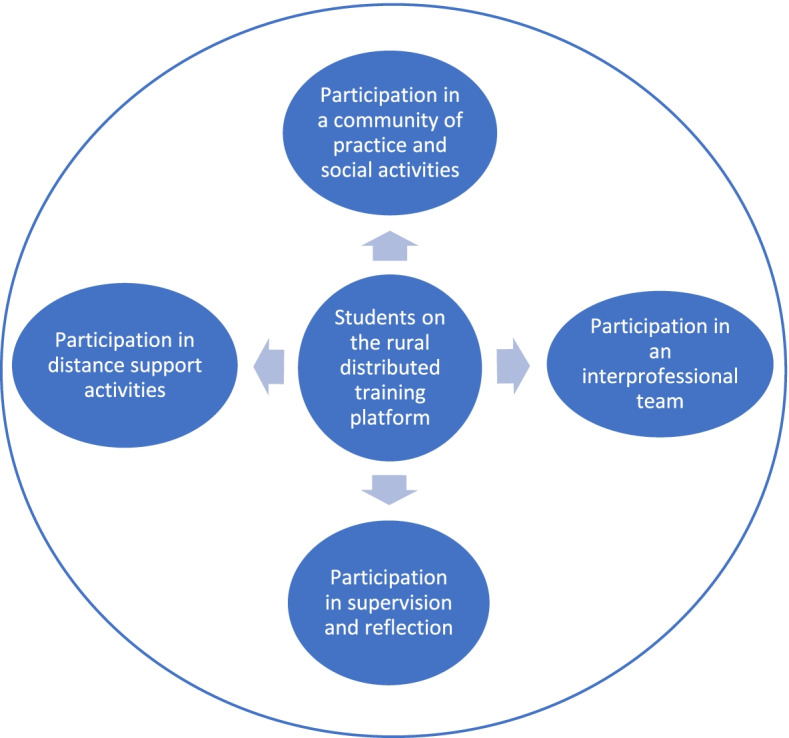
The suggested extent of student participation on rural clinical training platforms

The findings highlight not only the importance of students’ participation in the place they are training or in the rural community of practice, but also the need for active participation in supervision and reflection and in academic and social support to facilitate transformative learning. This is corroborated by the recent editorial in Medical Education by Badawy et al. (2021) [31] who emphasize the importance of social networks. The participation of the students in these various activities and practices are depicted in Fig. 1.

Learning is complex and continuously evolving and requires support and facilitated reflection to allow students the opportunity to truly benefit from uncomfortable learning experiences. It is precisely the variability in individual experiences within a context that provides opportunities for transformation through active participation that we believe highlights the personal dimension of transformative learning.

Critical reflection is foundational to Mezirow’s transformative learning theory and educators can play a significant role in facilitating such reflection for students [28]. Some students responded positively to distance support, which facilitated critical reflection and allowed students to process challenging issues in the clinical context. Considering the resource challenges with competing demands for local clinicians, we propose that this function could be fulfilled by academic staff from the training institution, who may support critical reflection remotely. In our experience critical reflection can be done using written narratives, reflective interviews or case-based discussions utilizing various distance communication and video-conferencing tools. Facilitating critical reflection from a distance, as a supplementary strategy at rural distributed sites, appears to be a necessary addition to programmes.

Evident in this data was the notion that the support needs of students extended beyond the academic and into the personal, emphasizing the importance of a holistic understanding of learning. Here too we see that the experience of students varied at an individual level. Students on short rotations appear to have enjoyed a greater sense of belonging and social inclusion than their counterparts placed at this site for the year. At face value this seems surprising, as one might expect that being immersed within a context for a longer period would provide greater opportunity for social inclusivity. Social isolation is often however, only felt over a longer period, once a person has settled into a new environment and may be experiencing some of the irritability of culture shock. It is not easily noticed in the excitement of a shorter term placement when students may be in the euphoric stage of culture shock [32, 33]. Perhaps the experience of social isolation in part reflects the variability of students’ clinical exposure, both in nature and location, especially as it relates to interprofessional engagement. Interprofessional collaboration between rehabilitation professions, it seemed, was deeply entrenched within the organizational culture at DHS Hospital, which is true of many rural health services [34].

Students on short rotations in this cohort, experienced greater levels of interprofessional engagement both clinically and socially, because interprofessional clinical learning activities were explicitly scheduled for these students. Engagement within these activities were however self-determined by long-term medical students who did not benefit from the interprofessional socialization. The long-term medical students were allocated to specific clinical disciplines and may have been expected to work longer hours, which influenced their opportunities to interact with other students. Additionally, the rehabilitation department which housed the short rotation rehabilitation students is, by geographical construct a multidisciplinary space, which may have more naturally facilitated greater levels of social inclusivity. It has been proposed that rural environments lend themselves more naturally to cooperative interaction between professions and in so doing, create an enabling learning environment [24].

Interprofessional socialization is a well-known aspect of improving students’ knowledge and collaboration between professions and should be encouraged in all sectors [35]. Traditional hierarchies in the workplace often contribute to professional groups sticking together. Providing a ‘base’ for students from all professions to engage with one another and other professions to develop relationships and trust in a collaborative environment, could add value to their social and professional development [34]. From the findings of this study, it appears that interprofessional socialization may be a useful tool to provide opportunities for developing social support structures for students doing longitudinal distributed training.

Coordination related to access, not only to academic material but also social support networks from a remote setting, was crucial to the students’ experiences on the distributed training platform. Feelings of isolation and of being negatively affected by missing academic tutorials is a very real experience for students and every effort needs to be made to ensure that students remain connected to their class and their support systems when they are based so far from their academic home [31]. An understanding of why students who were placed in Upington for the entire year seemed to feel more socially isolated is necessary to better support these students and encourage the development of local support systems.

The findings from this study highlight not only the importance of students’ participation in the place where they are training or in the rural community of practice, but also the need for active participation in supervision and reflection and in distance academic and social support initiatives to facilitate transformative learning [13, 29]. Intentional coordination of opportunities for students to create and sustain relationships with peers on the distributed clinical platform in order to build trust and emotional support networks is imperative [36]. There is an evident need for planning and preparing future distributed training sites with a focus on academic and social support structures being in place prior to the students' arrival.

## Conclusion

In this paper we strived to explore the experiences and perceptions of student learning on a new distributed rural training platform from the perspective of different stakeholders. The three dimensions and interrelationships of person (student, clinical educators and remote academic staff), place (distributed rural placement) and action/participation (supervision; reflection and support) are vital building blocks to facilitate transformative learning on the rural platform. The dimension of participation can however be further unpacked to include the four suggested areas of participation as depicted in Fig. 1. Not only is participation in a rural community of practice necessary, but also within interprofessional teams, in supervision and reflection and in distance academic and social support. These interrelationships need to be nurtured in the long term for clinical exposure to be relevant and responsive to the needs of the students, the context and the people they serve.

## Data Availability

Interiew schedules and surveys generated during the research process can be found in the following publication: Muller J, Reardon C, Hanekom S, Bester J, Coetzee F, Dube K, et al. Training for Transformation: Opportunities and Challenges for Health Workforce Sustainability in Developing a Remote Clinical Training Platform. Front Public Heal. 2021;9 April:1–14. All data related to analysis for the current study are not publicly available due to reasons of participant confidentiallity, but are available from the corresponding author on reasonable request.

## Abbreviations

APM: Academic programme managers
DHS: Dr Harry Surtie Hospital
FMHS: Faculty of Medicine and Health Sciences
HFM: Dr Harry Surtie Hospital facility managers
MBChB pre/post: Short rotation medical students
MS: Longitudinal integrated clerkship medical students
NSC: Non-supervising clinicians and staff
OT pre/post: Short rotation Occupational Therapy students (pre/post rotation)
PHC: Primary health care
PT pre/post: Short rotation Physiotherapy Students (pre/post rotation)
REDCap: Research Electronic Data Capture (™)
